# Perspectives of Zambian Clinical Oncology Trainees in the MD Anderson and Zambia Virtual Clinical Research Training Program (MOZART)

**DOI:** 10.1093/oncolo/oyac110

**Published:** 2022-06-11

**Authors:** Kevin Diao, Darya A Kizub, Noveen Ausat, Catherine K Mwaba, Chidinma P Anakwenze Akinfenwa, Carrie A Cameron, Elizabeth Y Chiao, Dorothy C Lombe, Susan C Msadabwe, Lilie L Lin

**Affiliations:** Department of Radiation Oncology, The University of Texas MD Anderson Cancer Center, Houston, TX, USA; Department of General Oncology, The University of Texas MD Anderson Cancer Center, Houston, TX, USA; Department of Radiation Oncology, The University of Texas MD Anderson Cancer Center, Houston, TX, USA; Department of Oncology, Cancer Diseases Hospital, Lusaka, Zambia; Department of Radiation Oncology, The University of Texas MD Anderson Cancer Center, Houston, TX, USA; Department of Behavioral Science, The University of Texas MD Anderson Cancer Center, Houston, TX, USA; Department of Epidemiology, The University of Texas MD Anderson Cancer Center, Houston, TX, USA; Department of Radiation Oncology, MidCentral District Health Board, Palmerston North, New Zealand; Department of Oncology, Cancer Diseases Hospital, Lusaka, Zambia; Department of Radiation Oncology, The University of Texas MD Anderson Cancer Center, Houston, TX, USA

**Keywords:** clinical research, training program, virtual, oncology, international, Africa

## Abstract

**Background:**

African countries are underrepresented in cancer research, partly because of a lack of structured curricula on clinical research during medical education. To address this need, the MD Anderson and Zambia Virtual Clinical Research Training Program (MOZART) was developed jointly by MD Anderson Cancer Center (MDA) and the Cancer Diseases Hospital in Zambia (CDH) for Zambian clinical oncology trainees. We explored participant perspectives to provide insight for implementation of similar efforts.

**Materials and Methods:**

The MD Anderson and Zambia Virtual Clinical Research Training Program consisted of weekly virtual lectures and support of Zambian-led research protocols through longitudinal mentorship groups that included CDH faculty and MDA peer and faculty mentors. Participants were contacted via email to take part in semi-structured interviews, which were conducted via teleconference and audio-recorded, transcribed, and coded. Emergent themes were extracted and are presented with representative verbatim quotations.

**Results:**

Thirteen of the 14 (93%) trainees were interviewed. Emergent themes included (1) participants having diverse educational backgrounds but limited exposure to clinical research, (2) importance of cancer research specific to a resource-constrained setting, (3) complementary roles of peer mentors and local and international faculty mentors, (4) positive impact on clinical research skills but importance of a longitudinal program and early exposure to clinical research, and (5) challenges with executing research protocols.

**Conclusion:**

To our knowledge, this is the first qualitative study of African clinical oncology trainees participating in a virtual clinical research training program. The lessons learned from semi-structured interviews with participants in MOZART provided valuable insights that can inform the development of similar clinical research training efforts and scale-up.

## Teaser

African countries are underrepresented in cancer research, partly because of a lack of structured curricula on clinical research during medical education. To address this need, the MOZART program for Zambian clinical oncology trainees was developed jointly by the MD Anderson Cancer Center (MDA) and the Cancer Diseases Hospital in Zambia (CDH). This article explores participant perspectives to provide insight for implementation of similar programs.

**Implications for Practice**

The MD Anderson and Zambia Virtual Clinical Research Training Program is a fully virtual, international partnership to provide clinical research training for Zambian clinical oncology trainees. Participants’ perspectives were studied through semi-structured interviews. Participants expressed both interest in and understanding of the need for clinical research specific to their resource-constrained practice environments. International peer mentorship was uniquely beneficial and may increase the capacity for research mentorship. Challenges encountered in learning about and performing clinical research demonstrated the importance of a longitudinal program and early exposure to clinical research. These lessons can be applied to the implementation and scale-up of similar efforts throughout Africa.

## Introduction

African countries are experiencing a rapid increase in cancer burden but are underrepresented in cancer research for numerous reasons, including a lack of structured training in clinical research during medical education.^1^ Training local physicians in clinical research methods is a potentially scalable and sustainable solution to the shortage of clinical research productivity.^2^ The development of virtual educational partnerships between academic medical centers with strong clinical research infrastructure and resource-constrained centers in low- and middle-income countries (LMICs) will be important to address this need, especially as models of collaboration shift to a virtual format as an adaptation to the COVID-19 pandemic.^3^ Building on an existing academic partnership,^4^ The University of Texas MD Anderson Cancer Center (MDA) and the Cancer Diseases Hospital in Zambia (CDH) developed a joint virtual clinical research education program for Zambian clinical oncology trainees, known as the MD Anderson and Zambia Virtual Clinical Research Training Program (MOZART). This program consists of weekly virtual lectures from content experts at MDA and CDH and support of Zambian-led research protocols through longitudinal mentorship.

Virtual instruction in clinical research for African clinical oncology trainees may increase training capacity and longitudinal partnerships while reducing costs associated with implementation, but the benefits and challenges of such a program have not been explored from the participants’ perspective, which should be considered when designing an effective curriculum. International peer mentorship may be an important component of expanding clinical research mentorship capacity, but differences in peer versus faculty mentorship and local versus international mentorship have not been studied in this context. This information could be uniquely valuable in designing and improving future clinical research training initiatives in Africa. In this study, we explored the perspectives of participants in MOZART through semi-structured interviews with the goal of refining the program and providing recommendations for implementation and scale-up of similar efforts.

## Materials and Methods

### Program Structure

**Figure 1.**
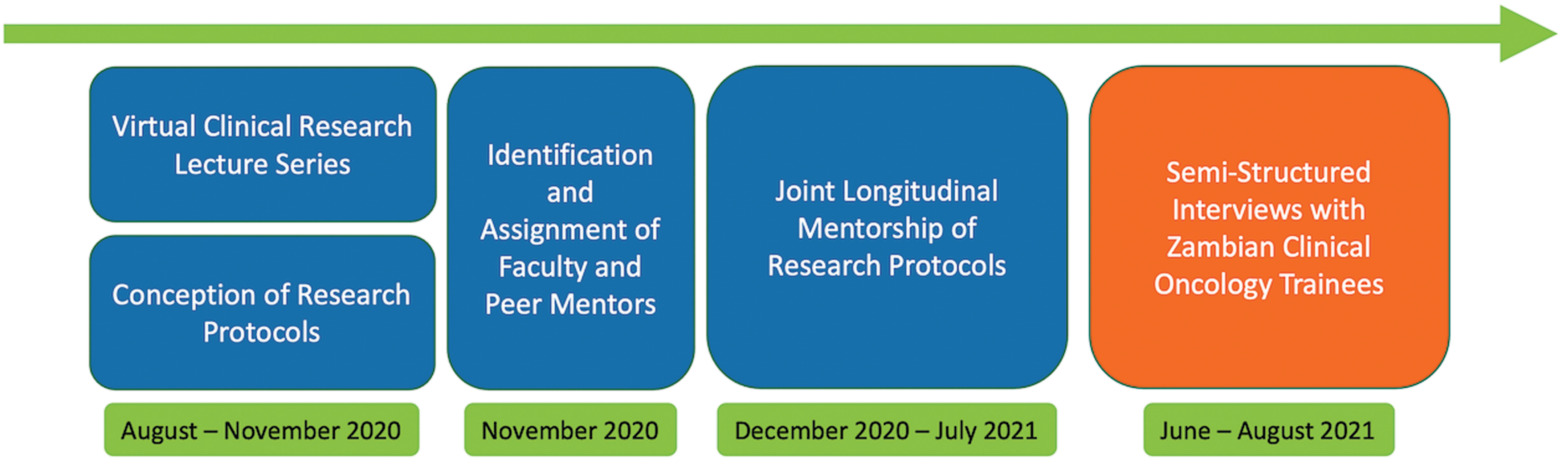
Schematic timeline of the MD Anderson and Zambia Virtual Clinical Research Training Program (MOZART).

The first group of trainees in the CDH clinical oncology program enrolled in this 4-year program in 2018. All CDH clinical oncology trainees were required to attend MOZART as this was integrated as a formal part of their training curriculum. There was no competitive application process. The pilot clinical research training program took place between August 2020 and July 2021. The didactic curriculum included lectures on developing mentor relationships, identifying a research question, evaluating a research paper, research types and study design, institutional review boards (IRBs) and research ethics, biostatistics, scientific writing, and clinical trial design, among others. This was followed by a period of longitudinal research mentorship (Fig. 1).

The trainees initially identified an area of research interest with their local CDH faculty mentor and were thereafter paired with peer resident or fellow mentors and faculty mentors at MDA. A total of 6 peer mentors and 6 international faculty mentors from MDA participated. The peer mentors were selected based on interest in global health, mentorship, and prior clinical research experience and all of them had at least one first author, peer-reviewed publication. Trainees and mentors were encouraged to meet at least once every 3 weeks via teleconference.

### Study Design

To explore participants’ perspectives about the program, we used a qualitative study design. The study was approved by the IRB at MDA. All participants were contacted via email to take part in a semi-structured interview and provided verbal informed consent to participate. Participants were not offered compensation. The semi-structured interviews were conducted via teleconference between June 2021 and September 2021 by a single member of the team (N.A.) from MDA who had not participated in the lecture series or previously interacted with the participants. With the permission of the interviewees, the interviews were audio recorded and manually transcribed. Data collection ended when all participants had been interviewed or declined to be interviewed.

The semi-structured interview guide was developed with input from both CDH and MDA investigators (Table 1). Thematic analysis of the transcripts from these interviews was used to examine patterns and relationships between themes and subthemes with the assistance of the qualitative data analysis software ATLAS.ti (version 9, Berlin, Germany). Each transcript was double-coded by 2 co-authors (K.D., D.A.K.) with the interview guide, and any differences were resolved through discussion.^5,6^ Emergent themes were extracted and are presented below, with representative verbatim quotations.

**Table 1.** Semi-structured interview guide.

A. Demographics
1. What year in your training program are you?
2. What is your primary spoken language?
3. What is your primary written language?
4. What country were you born in?
B. Prior clinical research experiences
1. Can you tell me about your educational background?
2. How would you describe your familiarity with clinical research prior to the clinical research course?
3. Can you tell me about any specific previous experiences with clinical research before taking part in the clinical research course?
C. Experience with lectures
1. In general, when you go to a lecture that’s very helpful to you, what is that like?
2. What did you hope to gain from participating in the clinical research course?
3. How do you think clinical research will be relevant to your future career?
4. Can you describe your experience participating in the lectures?
5. What topics do you feel needed either more or less attention during the course?
6. In what ways should the registrars engage with the material?
D. Application of course to research
1. How did the course prepare you to conduct your own clinical research?
2. What are some specific examples of how you applied what you learned in the course to your own research?
3. In what ways did you feel unprepared to conduct your own clinical research?
4. What aspects of the course were not as helpful?
5. How could these aspects be made more relevant for you?
E. Experience with mentorship
1. What are the characteristics of good mentorship relationships, in your opinion?
2. Tell me about your mentorship experience in the program.
3. How have your mentorship relationships helped you to develop your research protocol?
4. What challenges have you experienced along the way?
5. What things could a mentor do to help you with that challenge?
6. In what ways was your relationship with your peer mentor and faculty mentors different?
7. How did you view their roles as part of your research team?
8. How do you view the different role and relationships you have with your local and international (MD Anderson) mentors?
F. Conclusions
1. What do you think other oncology trainees should know about this course?
2. Can you explain why you would or would not recommend the course?
3. Would participating in the clinical research course again next year be useful?
4. Is there anything else that you would like to comment on about the clinical research course that we have not discussed today?

## Results

All 14 CDH clinical oncology trainees participated in MOZART, and of these, 13 (93%) agreed to be interviewed. Of the 13 interviewees, 7 were women and 6 were men, and the median age was 34 years (range, 30-49). Five were in their 2nd year, 4 third year, and 4 were in their fourth year of training. Primary languages spoken were English (8) and other languages (9) including Bemba, Nyanja, Tok Pisin, Sotho, Pidgin English, and French. Primary written languages were English (12) and other languages (3) including Nyanja, Sotho, and French. Birth/home countries were Zambia (7) and other countries (6) including Sierra Leone, Malawi, Papua New Guinea, Lesotho, and Democratic Republic of the Congo. The average number of virtual meetings per mentorship group was 10 meetings over 8 months. However, mentorship was also provided outside of planned meetings such as via email and mobile apps, and peer mentors estimated that they provided an average of 18 h of mentorship time over 8 months. Zambian fellow-led research project titles are summarized in Table 2.

**Table 2.** Zambian fellow-led research project titles.

Assessing the patterns of presentation and management of pediatric brain tumor patients at the Cancer Diseases Hospital.
Epidemiological and clinical characteristics of oropharynx cancer at Cancer Diseases Hospital.
Comparing the role of induction chemotherapy followed by concurrent chemoradiotherapy versus chemoradiotherapy alone in the treatment of patients with locally advanced nasopharyngeal cancer at Cancer Diseases Hospital.
Retrospective review of conjunctival squamous cell carcinoma at Cancer Diseases Hospital.
The effect of delaying radiotherapy on local recurrence and survival in patients with stage III Wilms Tumor at Cancer Diseases Hospital.
Retrospective evaluation of toxicity outcomes for 2D versus 3D planning for locally advanced cervical cancer brachytherapy.
Measuring the overall benefit of 3D planning for breast radiotherapy in a resource-limited environment.
Overall survival and outcomes in geriatric cancer patients at Cancer Diseases Hospital.
Retrospective study to evaluate the causes of mortality for cervical cancer patients during chemoradiotherapy and within the acute period of treatment completion.
A retrospective study of de novo metastatic breast cancer incidence and overall survival: Does sociodemographic play a role in late presentation?
A retrospective study to establish why pediatric patients with retinoblastoma present with advanced disease at Cancer Diseases Hospital.
The influence of time from preoperative chemoradiation to surgery on rectal cancer patients at Cancer Diseases Hospital.
Corrigendum of FIGO staging and its impact on management of cervical carcinoma at Cancer Diseases Hospital.

Emergent themes from interviews included (1) diverse educational backgrounds but lack of exposure to clinical research, (2) the importance of cancer research specific to a resource-constrained setting, (3) complementary roles of peer mentors and local and international faculty mentors, 4) positive impact on clinical research skills but importance of a longitudinal program, and (5) challenges with research protocol execution.

### Diverse Educational Backgrounds but Lack of Exposure to Clinical Research

Many of the trainees in this program completed their medical training in countries other than Zambia, such as China, Cuba, Russia, Sierra Leone, Democratic Republic of the Congo, Malawi, and Papua New Guinea. Some started the clinical oncology program immediately after completing their internship and rural postings, but others worked as practicing physicians for more than a decade before applying to the program.

Despite these differences in educational background, trainees had not been exposed to clinical research curricula in their prior programs, and mostly lacked experience in participating in clinical research projects. One trainee had published a peer-reviewed case report as a middle author. One of the main reasons identified for the lack of exposure to clinical research was a focus on clinical education during prior training and a lesser emphasis on research in general medicine than in oncology.

There was a study that I participated in, as in it was just mostly data collection and things like that. It was not really the depth of the whole research and the writing, but mostly just assisting with data collection.

I wouldn’t say much experience, because during internship we don’t do anything of the kind, it’s more of like practicing [medicine]… So when I came to oncology that was the best experience with actually doing research. I would say I never did any until now when I came to clinical oncology.

For most of the trainees, participating in MOZART was both their first exposure to a clinical research curriculum and their first experience participating in a clinical research project.

I just didn’t know much about research and things like that because really it wasn’t part of our program in school during undergrad. There was nothing, we didn’t have courses on clinical research.

### Importance of Cancer Research Specific to a Resource-Constrained Setting

All the trainees felt that clinical research was important for a variety of reasons, including the need for systematic investigation of therapies, communication of scientific findings, advancement of medicine, ability to influence policymakers and policy implementation, and ultimately better patient care. However, the most mentioned reason was that existing published research is heavily skewed toward high-income countries and does not necessarily represent resource-constrained practice environments and particular patient populations. The trainees recognized that they did not have access to certain standard-of-care treatments, and research and evidence to guide their clinical decision-making when it deviated from established norms was lacking.

Yes, I think it will be so interesting [to do my own research]. For me I really want to know what exactly is happening because the conditions are different, us being in a low resource environment we don’t have this or have that and the standard protocol has everything. So it would be helpful to know what is really working and what is going on in our resource-constrained environment.

We really need clinical research because most of the time, I’ll speak for Zambia, we don’t have much research. We rely on data that is in the Western world, which may not necessarily be the same for us because we have different experiences. It’s important that we have research and I would like to be part of research so we can answer questions and be able to manage our patients better.

Trainees who were born in countries outside Zambia were motivated by a strong desire to bring their clinical research skills back to their home countries, which typically had very little cancer care infrastructure. They expressed excitement at the prospect of being able to generate and apply data from patients in their own countries to advance patient care locally and were eager to be among the first do so.

At the moment we only have 3 practicing clinical oncologists in [my country], so there are a lot of things that we don’t know about our own population that need a lot more research… Most of it, we extrapolate from data from neighboring countries. We are going to need this information even in my own career.

[My country] does not have a cancer center. We are going to be the first team to establish a cancer facility in [my country], so it means that it is a fertile land on which we can learn more on clinical research. So the experience in research would be for me a very important component in my training so that when I move back home I should be in a position to contribute to knowledge in the continent and in the field of research.

### Complementary Roles of Peer Mentors and Local and International Faculty Mentors

The trainees described a good mentor as someone who is accessible, has open lines of communication, sufficient time to spend with the trainee, and can provide material resources and guidance for their research projects. The trainees were particularly positive about the impact of the close working relationships they had developed with their MDA peer mentors. They spent the greatest amount of time working one-on-one with their peer mentors, who they found to be more accessible because of their somewhat less busy schedules and more open lines of communication through mobile devices. With the support of their peer mentor, the trainees would work on their research projects and then at longer intervals convene with the entire mentorship team, including faculty mentors, for additional feedback and guidance.

The peer mentor relationship was very good and accessible because we even set up a WhatsApp to which we could drop information to say can we have a meeting tomorrow. It was not too complicated, not too formal. We would have our discussions first even before the consultants join our meeting, like “correct this” or “correct that”.

I feel like the peer mentor would understand me. The peer mentor probably would have more time to have calls almost all the time as compared to my faculty mentors. My faculty mentors are busy people so I don’t think they have as much time as I would want with them.

[My peer mentor] has been there for me from the start. At times she even asked about my personal life. I had COVID and during that time she was even communicating with me asking how I was doing.

The trainees also found it helpful to have a local faculty mentor with whom they could interact with quickly and in person, and who also understood the complexities and feasibility of doing research in their system. The MDA faculty mentors provided guidance on how to ask important and interesting research questions and refining project aims and methods.

With my local mentor, the fact that she’s here within even as I’m doing the work. Sometimes I just pitch in and I have a question in mind I actually just get to ask her. So for the local mentor it’s easier, she’s always there.

My international mentor is able to give me a broader aspect as to how I can go about things and it marries in with my local mentor. My local mentor has the experience of some of the limitations here that I may not know of. While getting an idea from my international mentor, which is great, my local mentor will build on that or be able to tell my international mentor ‘this is not feasible in my environment’, or ‘we could do this’, so it’s important to have both.

### Impact on Clinical Research Skills and Importance of a Longitudinal Program

The trainees felt that a research curriculum was just as essential to their training as the clinical curriculum. They described a stark contrast in their clinical research preparedness before and after participating in MOZART, developing an appreciation for the structured process of research through the program.

I think they need to protect [ie, maintain and support the clinical research training program]. I would recommend that everyone take this. It’s a great opportunity, it builds confidence. It’s a platform for one to stand on their feet in research.

Going into the course I was naïve, but after the course I was able to carry out my dissertation project. I was able to find a topic to write on and how to go about getting permission to carry out the research.

It was a clear step by step process that helped us understand how to develop these research questions and how to take it all the way through the methodology, results, and how to analyze them into a compete research paper.

However, trainees were realistic in recognizing that much remained to be learned, and they felt that either a longer course or participating again would fill in some of the persistent gaps in their knowledge. Specifically, they found their training in biostatistics to be inadequate, as it consisted of only a single lecture. The more senior trainees wished that the curriculum had been implemented earlier during their training.

I feel obviously it helped because it went from not knowing anything to something, but I feel like I would still need more lectures to fully get exactly what research is.

There are some parts that I felt needed more light especially where it talked about how to calculate the sample size. There are some parts where you need the help of the biostatistician, but even before they come in at least we should be able to understand these issues, such as how to interpret results and how do you calculate the p-value.

I wish I would have had this [research course] in my first year, I think it would have helped me develop my research topic earlier and I would have even gotten a better understanding if this was something that I was coming back to every now and then… But that is something that people in the other years will get to experience.

### Challenges With Execution of Research Protocols

The trainees noted several challenges in progressing with their research protocols. They were balancing a full clinical load and exams and felt that they needed more protected time for research. Many of the trainees had experienced personal illness including contracting COVID-19 and were dealing with prolonged recovery from their ailments. Performing literature reviews was difficult without access to paid subscription journals. Data retrieval was also a barrier, because medical records are handwritten, often lack a uniform standard of documentation, and are prone to being lost in storage.

That for me has been the biggest challenge, finding time to actually sit and work on my research. If we could get even a free 2 hours at work where it is research time where you can just sit [and work on research].

I got COVID so during that period it was very difficult for me to actually meet with my mentor. So I lost some time from my mentoring situation.”

The main challenge I’m facing right now is getting data. There was a switch from the main hospital to the cancer hospital for the pediatric patients, and during that shift most of the data was misfiled… So we had to change the time period for my research.

Because I had thought the process had taken just too long, the lack of communication on my part may have breached my relationships with the peer mentor… maybe the part that I was supposed to do was to keep telling them that I have challenges collecting the information, it’s difficult to find the files. Reporting the same thing every day was getting to be a challenge. But we ended up going ahead with the information that we could collect.

## Discussion

Semi-structured interviews with participants in MOZART provided valuable insights into the scope of the trainees’ educational backgrounds and their plans for incorporating research into their future careers, the need for research conducted in a resource-constrained environment, the unique roles held by different types of mentors (peer vs. faculty, local vs. international) in professional development, the importance of a longitudinal training program with early exposure to clinical research, and challenges associated with conducting trainee-led research in their work environment. To our knowledge, this is the first qualitative study of the perspectives of African clinical oncology trainees participating in a virtual clinical research training program. The findings from this study can inform the development of similar clinical research training efforts and scale-up elsewhere.

Despite the diversity in medical education from countries around the globe, none of the participants in this study had received formal education in clinical research, and very few had participated in clinical research. This reflects an unmet need for clinical research training, including oncology-specific clinical research, in the region as supported by a recent survey of trainees and recent graduates of a Tanzanian clinical oncology program showing that only 23% reported prior research experience, 37% formal training in research methodology, and 13% research mentorship.^7^ The same study found that 87% of respondents intended to incorporate research into their future careers.

Our findings from the current study are similar. Because MOZART is a formal part of the Zambian clinical oncology curriculum, there was no competitive application process. Among this unselected group of trainees, there was a strong perception of the importance of clinical research. The trainees further perceived that existing published research is heavily skewed to high-income countries, the results of which do not necessarily represent or are relevant to local practice and patient populations. Trainees were highly motivated to pursue clinical research as part of their future careers to address this clear need, and those who came from countries with little cancer infrastructure were especially excited to be among the first to be performing clinical research when they returned to their home countries.

Limited faculty availability for mentorship has been described as a limitation of North-South partnership models, and other models such as facilitated mentorship where peers within a program mentor one another with the guidance of a facilitator have been developed in response.^8^ However, the use of international peer mentorship in the context of clinical research training is relatively untested. Having peer mentors from partner institutions with strong clinical research infrastructure may be particularly important for increasing mentorship capacity and should be further explored and leveraged. Some of the potential benefits of peer mentorship found here were better availability and approachability compared with faculty mentors, and the mutual benefit to the peer mentors of having global health opportunities that may shape their own career trajectories. The trainees in our program expressed high satisfaction with the relationships formed and time spent with their peer mentors. In a family medicine training program in Lesotho, international peer mentorship led to increased research confidence over time, but both the participants and international peer mentors themselves noted insufficient research expertise, highlighting the importance of a mixed-peer and faculty mentorship team and involving peer mentors with significant prior clinical research experience.^9^ Programs considering international peer mentors could consider entry criteria such as having peer-reviewed publications, prior mentoring experience, and/or mentorship training.

The trainees described unique challenges inherent to conducting research in resource-constrained environments, such as frequent personal illness, lack of high quality health data or access to journal articles, and insufficient time for research owing to high clinical demands.^10^ These underscore the importance of a longitudinal research program that accounts for these and other factors that tend to increase the amount of time required to learn clinical research skills and complete research projects, while “workshop” type single-instance initiatives may be less effective.

While these barriers to clinical research are complex, there are some potential solutions. Trainees in LMICs may have free access to a wide array of biomedical and health literature through Research4Life.^11^ A prospective breast and cervical cancer database of patients treated at CDH has been developed through external grant funding to CDH (PI: S.C.M.) and will be expanded through joint efforts. Providing protected research time for trainees is difficult given the inherent shortage of healthcare providers, but CDH leadership is committed to providing protected time apart from clinical duties for trainees to participate in MOZART. Some programs have hired general clinical associates to handle routine clinical tasks so that oncology trainees have dedicated research time.^12^

Other North-South research training collaborations include CARTA,^13^ ARCADE,^14^ WHO/TDR,^15^ and MEPI-MESAU,^16^ among others. For the most part, these programs focus on doctoral-level researchers, are not specific to oncology, and require a competitive application process. However, the success of these programs, which have improved progress on doctoral theses, peer-reviewed publication output, and success applying for grant funding demonstrates the potential impact of these types of partnerships. MOZART is novel and adds to these published experiences by providing a formal oncology-focused research curriculum within the CDH clinical oncology training program via a fully virtual platform, demonstrating the feasibility of increasing research mentorship capacity through the inclusion of international peer mentors.

One limitation of our study is that it was conducted at a single center in Sub-Saharan Africa, and the findings may not be generalizable to other African countries or LMICs. Nevertheless, participants in the program were from countries throughout Sub-Saharan Africa and responded similarly to the content, suggesting that the lessons learned are relevant in other settings. The training program described is still nascent, and the long-term impact on trainee research perceptions and development, career trajectory, and research productivity could not be captured. The interviews were conducted by an MDA team member who did not participate in the program or previously interact with the participants, but responses could have been biased to demonstrate an interest in research due to social desirability bias. Although our sample size was modest (*n* = 13), our response rate was high (93%) and data saturation was reached.^17^ The viewpoints reflected here are only of trainee participants, and future studies examining the perspectives of other stakeholders such as local and international mentors and institutional leaders may complement this data.

## Conclusion

Significant disparities still exist in cancer research output globally, despite a trend toward higher cancer burdens in LMICs, especially within Sub-Saharan Africa.^18^ Developing collaborative, virtual clinical research training partnerships between academic medical centers with strong clinical research infrastructure and resource-constrained centers will be important in the coming years to increase research capacity and address this gap, especially as COVID-19 has resulted in greater acceptance and feasibility of virtual partnerships.^19^ The perspectives of Zambian clinical oncology trainees participating in MOZART demonstrate a strong interest in clinical research, the unique and important role of international peer mentors as a potential source of increased mentorship capacity, the importance of longitudinal programs with early exposure of trainees to clinical research, and the challenges specific to performing clinical research as a trainee in a resource-constrained environment. The lessons learned can inform the development and scale-up of clinical research initiatives for other African clinical oncology trainees.

## Data Availability

The data underlying this article cannot be shared publicly for the privacy of individuals that participated in the study.
